# Formability Analysis of Bamboo Fabric Reinforced Poly (Lactic) Acid Composites

**DOI:** 10.3390/ma9070539

**Published:** 2016-07-02

**Authors:** Nurul Fazita M. R., Krishnan Jayaraman, Debes Bhattacharyya

**Affiliations:** 1 Centre for Advanced Composite Materials (CACM), Department of Mechanical Engineering, The University of Auckland, Private Bag 92019, Auckland 1142, New Zealand; k.jayaraman@auckland.ac.nz (K.J.); d.bhattacharyya@auckland.ac.nz (D.B.); 2 School of Industrial Technology, Universiti Sains Malaysia, Penang 11800, Malaysia

**Keywords:** bamboo fabric, defects, grid strain analysis, natural fibre fabrics, poly (lactic) acid, thermoforming

## Abstract

Poly (lactic) acid (PLA) composites have made their way into various applications that may require thermoforming to produce 3D shapes. Wrinkles are common in many forming processes and identification of the forming parameters to prevent them in the useful part of the mechanical component is a key consideration. Better prediction of such defects helps to significantly reduce the time required for a tooling design process. The purpose of the experiment discussed here is to investigate the effects of different test parameters on the occurrence of deformations during sheet forming of double curvature shapes with bamboo fabric reinforced-PLA composites. The results demonstrated that the domes formed using hot tooling conditions were better in quality than those formed using cold tooling conditions. Wrinkles were more profound in the warp direction of the composite domes compared to the weft direction. Grid Strain Analysis (GSA) identifies the regions of severe deformation and provides useful information regarding the optimisation of processing parameters.

## 1. Introduction

The use of Poly (lactic) acid (PLA) as a packaging material is limited due to several drawbacks such as low impact strength and brittleness [1,2]. Therefore, reinforcing PLA with natural fibre fabric can be one of the solutions to overcome its limitations. In this study, bamboo fabric has been used as reinforcing material in PLA composites. The formability of bamboo fabric-PLA composites has to be understood, since thermoforming is one of the major processes used to convert flat composite sheets into the packaging products [3].

Thermoforming technologies are currently being established for woven fabric-reinforced thermoplastic materials because these materials exhibit high impact strength, formability, shorter fabrication time and ease of handling [4]. The in-plane shearing properties for woven fabric-reinforced thermoplastic composites during processing must be carefully analysed to improve thermoforming production. When forming continuous fibre-reinforced materials, instabilities such as wrinkling and buckling may occur. In the case of 3-D forming using the matched-die thermoforming technique, the processing conditions can be optimised by individually investigating the parameters, which largely govern the properties of the finished component ultimately enabling the production of defect-free parts [5]. Die conformity of continuous fibre reinforced thermoplastics can therefore only be achieved through flow processes such as transverse flow, inter- and intra-ply slip and inter-ply rotation. The inter-ply rotation not only causes local changes in fibre orientation between adjacent plies but also leads to substantial thickness variations in the laminate [6]. These flow processes rely strongly on the laminate layup, the fibre-matrix combination, and the processing conditions such as pressure, temperature and forming speed, all of which have a great influence on the final part quality [7].

A well-established method of assessing the ability of a particular material to form 3-D components is through the production of hemispherical domes. Previous experimental work in this area has focused on forming hemispherical domes using either a matched-die or diaphragm forming with circular preforms [6,8]. A few articles have been published on the formability analyses of unidirectional, cross-ply and quasi-isotropic laminates [6,8,9,10]. Formability analyses of textile reinforced laminates have also been conducted by many researchers [11,12,13,14]. However, most of the formability studies have been conducted on petroleum based thermoplastic composites; very little work has been done on the formability of biopolymer based composites.

The purpose of the experiment discussed here is to investigate the effect of different parameters on the occurrence of deformations during sheet forming of double curvature shapes with bamboo fabric-PLA composites. The effect of unbalanced woven fabrics on the thermoformability of the composite laminate was studied. Further analysis was also carried out to investigate the behaviour of laminates with different blank sizes and different fabric stacking sequences. To obtain a physical measure of the specimen’s behaviour, the Grid Strain Analysis (GSA) technique was used to investigate the surface and thickness strains in the material.

## 2. Materials and Methods

### 2.1. Materials

**Figure 1:**
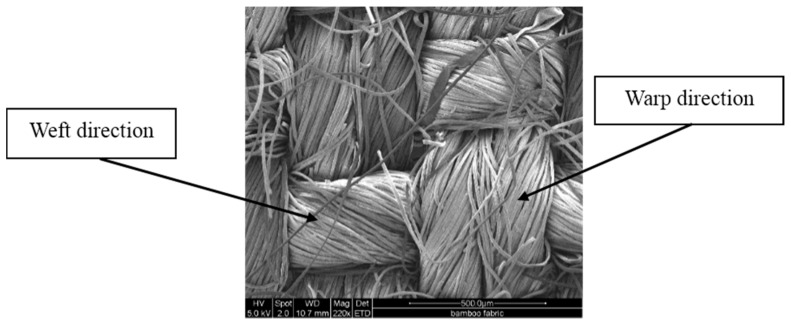
Environmental scanning electron microscopy (ESEM) image of the bamboo fabric [2].

Plain woven bamboo fabric, shown in Figure 1 (2 warps × 1 weft), was supplied by Industrial Textiles Limited, Auckland, New Zealand. The yarn thicknesses in the fabric were 50 tex in the warp direction and 71.4 tex in the weft direction while the fabric count was 104 × 56 per square inch. The areal density of the bamboo fabric was 230 g/m^2^. PLA (2003D) sheets of 0.25 mm thickness, manufactured by NatureWorks, were supplied by Alto Packaging Limited, Hastings, New Zealand. The mechanical, physical and thermal properties of the PLA (according to the datasheet) are shown in Table 1.

### 2.2. Fabrication of Composites

**Figure 2:**
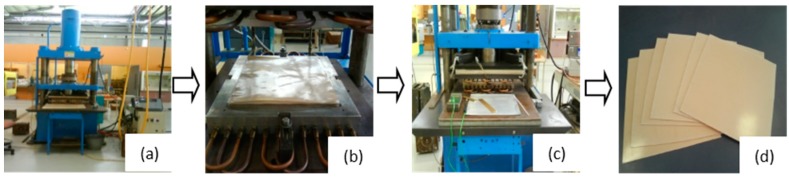
Manufacturing method for bamboo fabric-PLA composite sheets: (**a**) A square mould placed inside a 100 tonne hydraulic press, was heated using electrical and oil heaters; (**b**) Bamboo fabric-PLA stack was placed inside the mould; (**c**) The mould was closed without pressure for 2 min, followed by the application of 1.05 MPa pressure for 3 min; (**d**) Bamboo fabric-PLA composite sheets.

A square mould of dimensions 400 mm × 400 mm, placed inside a 100 tonne hydraulic press, was heated using electrical and oil heaters to 160 °C. A thermocouple was placed outside the mould in order to monitor its temperature. Once the mould reached 160 °C, the upper platen was opened and the bamboo fabric-PLA stack was quickly placed inside the mould. Then, the mould was closed without pressure for 2 min to allow permeation of the polymer through the fabric, followed by the application of 1.05 MPa pressure for 3 min. The temperature was held at 160 °C; after the set heating time, the mould was cooled by circulating cold water. The composite was kept under the set pressure until it cooled to 25 °C. The cooling rate was 2.25 °C/min. The platen was opened and the composite was removed from the press [2]. Figure 2 shows the simplified manufacturing method for the composites.

**Figure 3:**
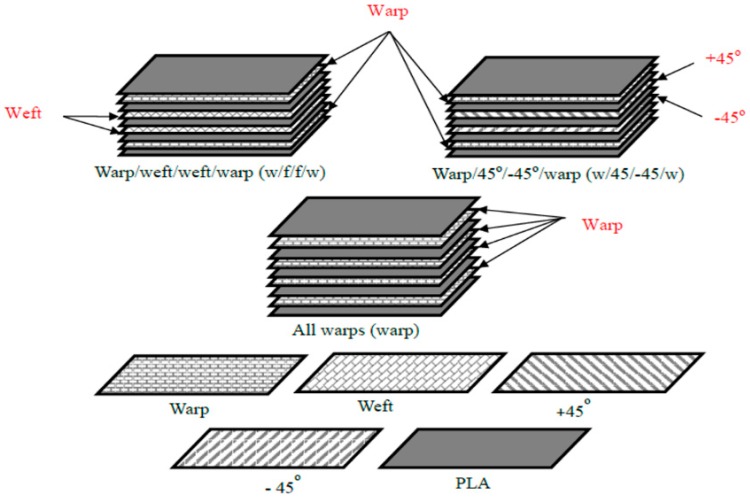
Schematic illustrations of laminates with various bamboo fabric stacking sequences.

Four layers of bamboo fabric and five layers of PLA sheets were arranged alternately with each other in a stack, as shown in Figure 3. This resulted in a fibre weight fraction of 0.35 (0.34 fibre volume fraction) and 1.8 mm thickness. The fibre volume fraction of the composites was calculated using Equation (1):

(1) $$V_{f}=\frac{\frac{W_{f}}{\rho_{f}}}{\frac{W_{f}}{\rho_{f}}+\frac{W_{m}}{\rho_{m}}}$$

where, *V_f_* = Fibre volume fraction, *W_f_* = Fibre weight fraction, *W_m_* = Matrix weight fraction, *ρ_f_* = Fibre density, *ρ_m_* = Matrix density.

In order to confirm the fibre volume fraction of the composites after manufactured, the yarns were extracted from the bamboo fabric-PLA composites by Soxhlet extraction, which was similar to the method prescribed in ASTM D 3171-11 standard [15]. The details on the Soxhlet extraction procedure can be found in the literature [16]. The fibre volume fraction for all bamboo fabric-PLA composite samples has been confirmed to be 0.34. Laminates with different stacking sequences, namely all warps (warp), warp/weft/weft/warp (w/f/f/w) and warp/45°/−45°/warp (w/45/−45/w), were manufactured as shown in Figure 3.

**Figure 4:**
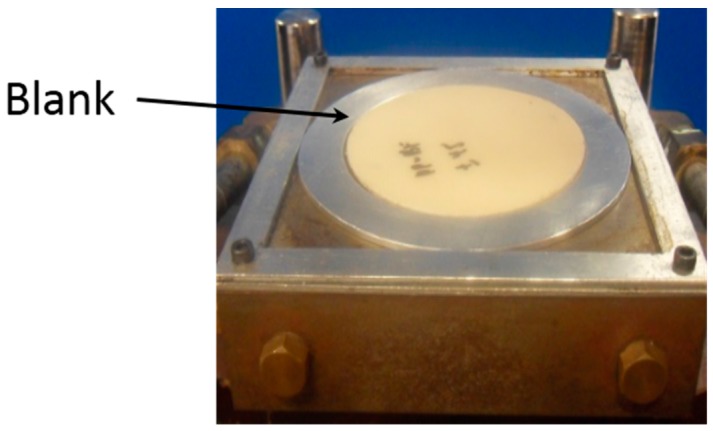
Blank from bamboo fabric-PLA composite laminate.

Circular blanks (Figure 4) for the forming experiments were cut from bamboo fabric-PLA composite laminates with warp, w/f/f/w and w/45/−45/w layups, in order to evaluate the effect of fabric orientations on the formability of the laminates. Five specimens have been tested for each different layups and conditions. A 2 mm rectangular grid was applied with a grid drawing machine to the top surfaces of the blanks. The grids were used to determine the deformations of the blank after forming using the GSA method.

### 2.3. Differential Scanning Calorimetry (DSC)

Bhattacharyya et al. [17] reported that thermoplastic composites can be formed at temperatures well below the melting point of the thermoplastic matrix, provided the specimens are heated above the melting point and formed before the matrix is able to reach its maximum degree of crystallinity. The formable temperature range (forming window) below the melting temperature of the matrix can be determined using differential scanning calorimetry (DSC). The glass transition temperature, melting temperature and crystallinity were determined using a DSC Q 100 (TA Instruments, New Castle, DE, USA). Analyses of the results were carried out with a TA Instruments Universal Analyser 2000 (TA Instruments, New Castle, DE, USA). Measurement was conducted on 9 mg samples in open aluminium pans under a nitrogen atmosphere at 45 mL/min flow rate, with a heating range 20–250 °C and a rate of 10 °C/min.

### 2.4. Matched-Die Forming Setup

Formability, using matched-die forming, was examined as a function of forming rate, die temperature, blank temperature, blank size and fibre orientation in the blank.

#### 2.4.1. Dome Forming Setup for Cold Tooling Conditions

**Figure 5:**
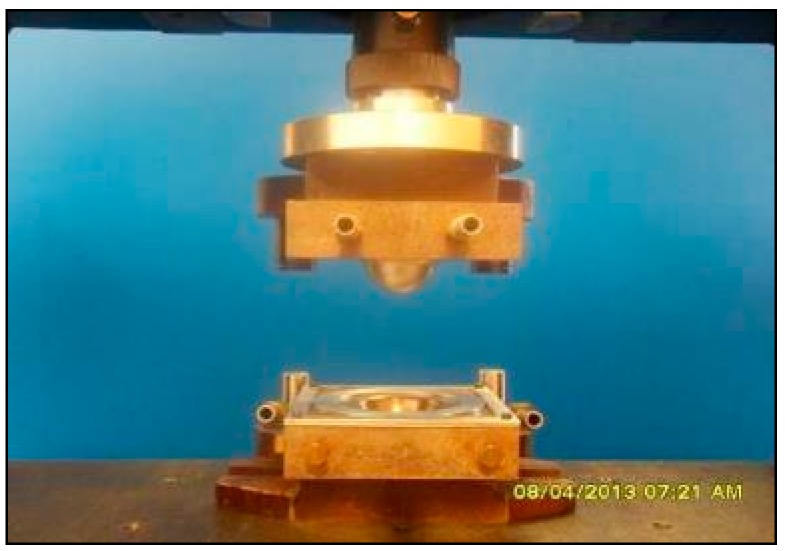
Dome forming setup using cold tooling conditions.

Figure 5 shows a photograph of the hemispherical dome forming setup. Table 1 lists the parameters for the experiments performed with bamboo fabric-PLA composites under cold tooling conditions. The dome forming equipment itself consists of matching, hemispherical male and female dies, with an inner radius of 25 mm, mounted in a universal mechanical testing machine (Instron model 1185, Canton, MA, USA). Specimens were heated to 155 °C by placing the samples on an insulating board in a convection oven and the sheet temperature was monitored using a K-thermocouple. Once the sheets reached 155 °C, they were removed from the oven and positioned upon the dome forming dies which were then set in motion. Once forming had been completed, the male die was withdrawn and finally, the formed part was removed from between the moulds. The cooling rate was 52.5 °C/min.

#### 2.4.2. Dome Forming Setup for Hot Tooling Conditions

**Figure 6:**
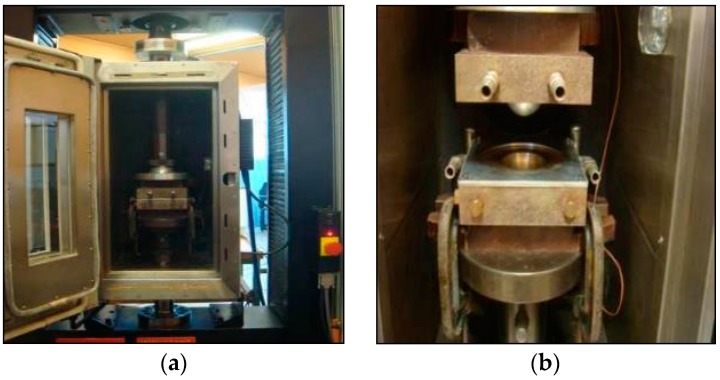
(**a**) Dome forming experimental setup and (**b**) close up of matched-die forming equipment.

Figure 6 displays a photograph of the hemispherical dome forming setup in the environmental chamber and a close-up of the matched-die forming equipment. The rig is capable of a number of different forming conditions including isothermal and non–isothermal conditions.

Table 2 lists the parameters for the experiments performed on bamboo fabric-PLA composites under hot tooling conditions. The tooling was fixed inside an environment chamber which was mounted in the testing machine (Instron model 1185, Canton, MA, USA) for temperature control of the blank and tool surfaces. The bottom half of the die was attached to the testing machine crosshead to allow forming rate control. Thermocouples were used to monitor the blank and die temperatures. Forming forces (which did not yield any significant information on the forming behaviour) could also be recorded.

### 2.5. Grid Strain Analysis (GSA)

In order to improve sheet forming operations, the deformation occurring in the laminate should be understood. However, by only observing the shape of the formed part and thickness variations in the laminate after forming, this information is difficult to gain. These observations cannot completely describe the deformation because in-plane strains may occur without changing the sheet thickness (pure drawing) during forming [18]. To obtain a physical measure of the specimen’s behaviour, the GSA technique was used to investigate the surface and thickness strains in the material.

GSA is a useful technique to gain a greater understanding into the sheet forming process since sheet forming procedures are often complicated and produce large strains in the deformed materials. GSA is an approach, which quantifies those strains by measuring the dimensions of the deformed grids and comparing them with the original grids printed on the material. In this method, the surface of a sheet is marked with series of square grids prior to deformation and the deformed shape of these grids is measured by measuring each element separately or by measuring the nodal coordinates of the deformed grids. In addition, the thickness changes in the sheet can also be reliably predicted, since the GSA method is capable of quantifying the deformation of the whole laminate, rather than just the surface layer. In the case of continuous fibre reinforced laminates, GSA can provide direct information on the deformation undergone by the surface ply only, but will also yield information on how sub-surface plies influence the surface layer behaviour. There are essentially two useful methods for presenting the strain distribution over the surface of a component, strain (arrow diagrams) and thickness contour maps [18]. The fundamental concept behind GSA and its application to components formed from continuous fibre-reinforced thermoplastic sheets has been presented by Martin et al. [19].

## 3. Results and Discussion

### 3.1. Differential Scanning Calorimetry (DSC) Results

**Figure 7:**
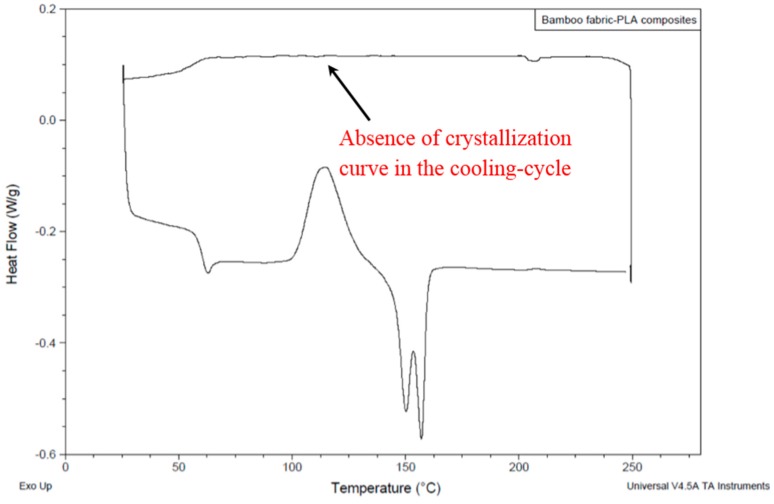
DSC trace of bamboo fabric-PLA composites.

The DSC trace of the bamboo fabric-PLA composites is shown in Figure 7. The forming window of bamboo fabric-PLA composites is difficult to determine from Figure 7, since the DSC trace did not show the crystallization curve in the cooling-cycle of the composites. No exothermic peak of crystallisation was found in bamboo fabric-PLA composites, which might be due to low molecular mass samples, as reported in Ahmed et al. [20]. Auras et al. [21] have reported that PLA normally has a narrow forming window (12 °C). In Figure 7, it is observed that the cold crystallization of bamboo fabric-PLA composites is within the temperature interval of 100 °C to 129 °C. The melting temperature of the composites is within the temperature interval of 130 °C to 163 °C. Therefore, experimental runs with different tooling conditions are required to determine the suitable forming temperature of the bamboo fabric-PLA composites.

### 3.2. Determination of Forming Parameters Using Different Tooling Conditions

#### 3.2.1. Bamboo fabric-PLA Composites Formed Using Cold Tooling Conditions

##### Forming Rate

**Figure 8:**
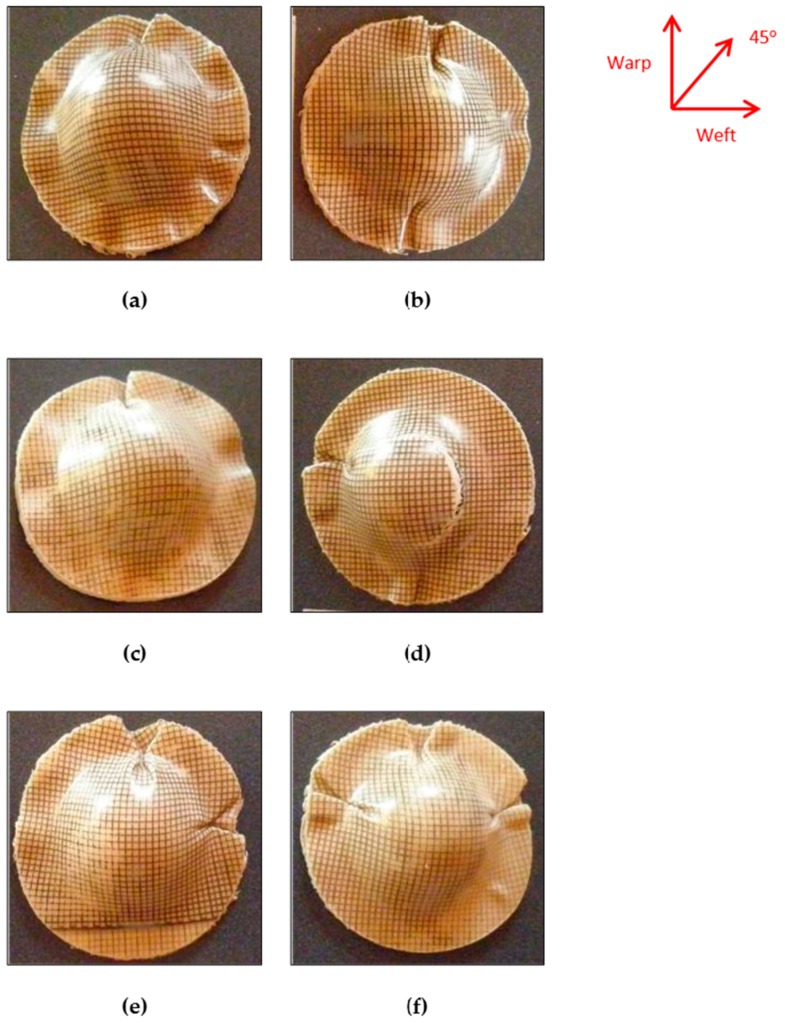
Bamboo fabric-PLA composite laminates with different fabric layups formed using cold tooling conditions at different forming rates. Blanks of 90 mm diameter were pre-heated to a temperature of 155 °C and formed at a die temperature of 23 °C. (**a**) w/45/−45/w, 100 mm/min; (**b**) w/f/f/w, 100 mm/min; (**c**) Warp, 100 mm/min; (**d**) w/45/−45/w, 60 mm/min; (**e**) w/f/f/w, 60 mm/min; (**f**) warp 60 mm/min.

As can be seen in Figure 8, the cold tooling conditions led to poor forming results for the bamboo fabric-PLA composites. With the 100 mm/min forming rate, the mould closing time is short and the forming temperature is relatively high. In this case, there is very little time for flow processes to occur, resulting in severe buckling at the flange area. However, with the lower forming rate (60 mm/min), breaking occurs at the apex region of the laminate for w/45/−45/w. This is the result of a low forming temperature preventing inter-ply slip from occurring. In this case, the shear stress acting on the plies does not exceed the shear yield stress of the matrix material, which ultimately results in fibre breakage at the apex region. Further, mould temperatures as low as 20 °C to 25 °C can cause “freezing” of the sheet and non-uniform drawing, as observed in other cases such as polyethylene terephthalate (PET) [22]. This implies that cold tooling conditions may not be a suitable forming method for PLA composites, likely due to its narrow forming window reported in literature [21]. Furthermore, the cooling rate during cold tooling condition was 52.5 °C/min. Behalek et al. [23] reported that samples cooled at rates higher than 10 °C/min could not crystallize and remained amorphous. It can be concluded that the forming temperature is a decisive parameter essentially influencing the quality of the forming results. It should therefore be well controlled and monitored throughout the thermoforming process.

#### 3.2.2. Bamboo Fabric-PLA Composites Formed Using Hot Tooling Conditions

##### Forming Temperature

**Figure 9:**
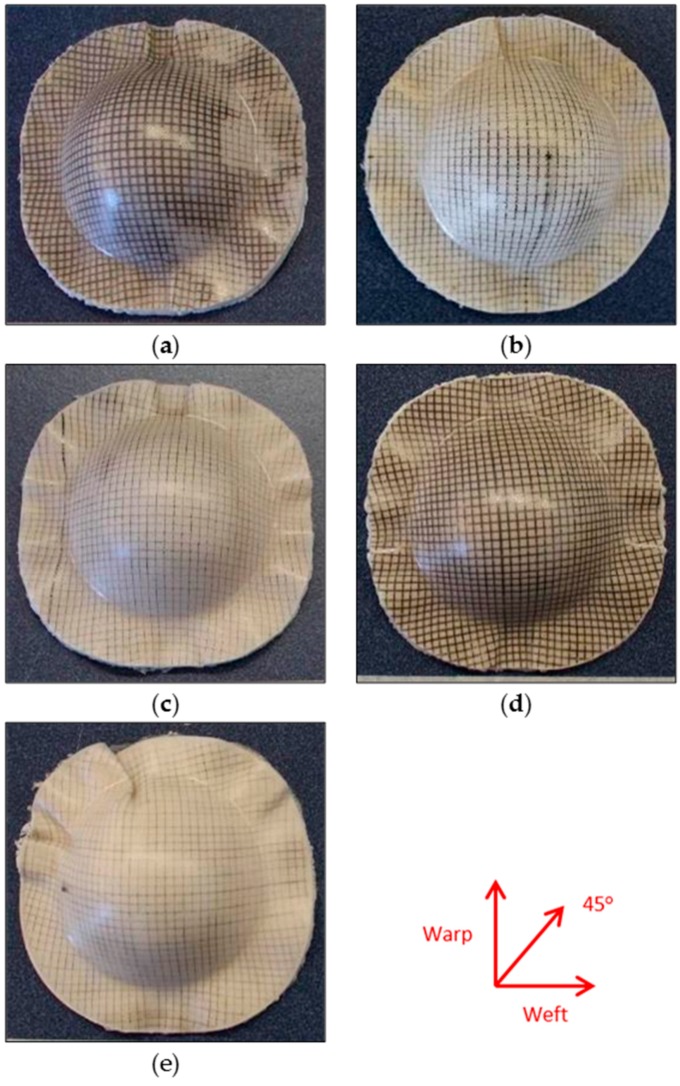
Bamboo fabric-PLA composite laminates with different fabric layups formed using blanks of 90 mm diameter using hot tooling conditions at different forming rates. (**a**) Warp, Die temperature = 130 °C, Blank temperature = 130 °C; Forming rate = 60 mm/min; (**b**) w/45/−45/w, Die temperature = 130 °C, Blank temperature = 130 °C, Forming rate = 60 mm/min; (**c**) w/f/f/w, Die temperature = 130 °C, Blank temperature = 130 °C, Forming rate = 60 mm/min; (**d**) w/f/f/w, Die temperature = 130 °C, Blank temperature = 130 °C, Forming rate = 40 mm/min; (**e**) w/f/f/w, Die temperature = 130 °C, Blank temperature = 140 °C, Forming rate = 60 mm/min.

Bamboo fabric-PLA composites formed using hot tooling conditions shown in Figure 9 indicate more promising results compared to those formed under cold tooling conditions. Laminates formed at 130 °C show less wrinkles compared to the laminates formed at 140 °C. If high forming temperature (in the melting temperature range) is applied, an unacceptably high level of fibre buckling is likely to occur. Figure 7 indicates that the melting temperature of the composites is within the temperature interval of 130 °C to 163 °C. At 130 °C, the viscosity of the PLA is considered adequate for flow processes to occur during the forming. Besides, when the sample was heated at temperatures close to the melting temperature of the PLA, crystals were partially melted. However, when the temperatures were too high (in the melting temperature range), the extremely high chain mobility hindered the regular arrangement of the polymer chain by the strain-induced crystallinity [24].

##### Forming Rate

For the same forming temperature of 130 °C, Figure 9 shows that laminates formed at the lower forming rate of 40 mm/min had a comparable quality with that of the laminates formed at 60 mm/min. Therefore, the lower forming rate was selected for the forming process as it will reduce the forming time. Furthermore, the formed PLA composite products exhibit good surface smoothness.

##### Blank Temperature

The study on the influence of different blank temperatures was performed using two different temperatures: 100 °C and 130 °C. According to Lim et al. [25], the thermoforming temperatures for PLA are in the range of 80–110 °C when the sheet enters the mould, which is much lower than other conventional thermoformed plastics such as polypropylene (PP).

**Figure 10:**
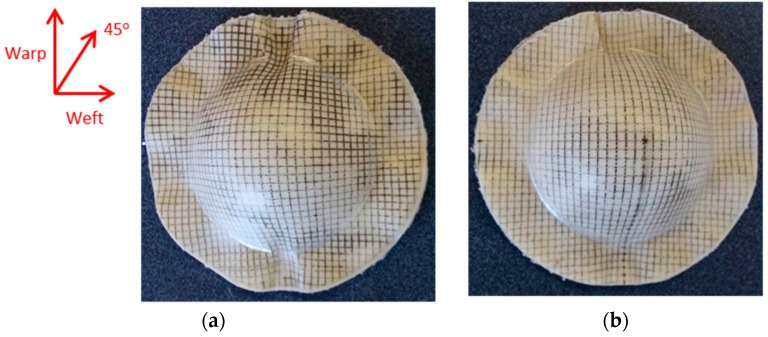
Bamboo fabric-PLA composite laminates of w/45/−45/w layup formed using different blank temperatures. (**a**) w/45/−45/w, Blank temperature = 100 °C, Die temperature = 130 °C, Forming rate = 60 mm/min, Blank size = 90 mm; (**b**) w/45/−45/w, Blank temperature = 130 °C, Die temperature = 130 °C, Forming, rate = 60 mm/min, Blank size = 90 mm.

It can be seen from Figure 10 that the w/45/−45/w dome formed using a 100 °C blank temperature appeared to be more elliptical with more wrinkles than the dome formed using a 130 °C blank temperature. Forming of the laminate at 100 °C was fairly successful but tended to result in wrinkles as at this temperature flow processes such as inter- and intra-ply slip are restricted. Although the die temperature was 130 °C, the laminate may not achieve this temperature during the forming process. Meanwhile, the domes produced with a 130 °C blank temperature were better formed as this temperature allows flow processes to take place within the laminate before cooling occurs. This is because the onset of melting temperature occurred at 130 °C.

### 3.3. Effect of Different Layups

In order to discuss the influence of different layups on the deformation of the bamboo fabric-PLA composite laminates, the blanks were thermoformed using a blank temperature of 130 °C, a die temperature of 130 °C and a forming rate of 60 mm/min. These parameters were selected based on the results obtained in Section 3.2. Using these particular forming parameters, the effects of different layups can be clearly observed.

**Figure 11:**
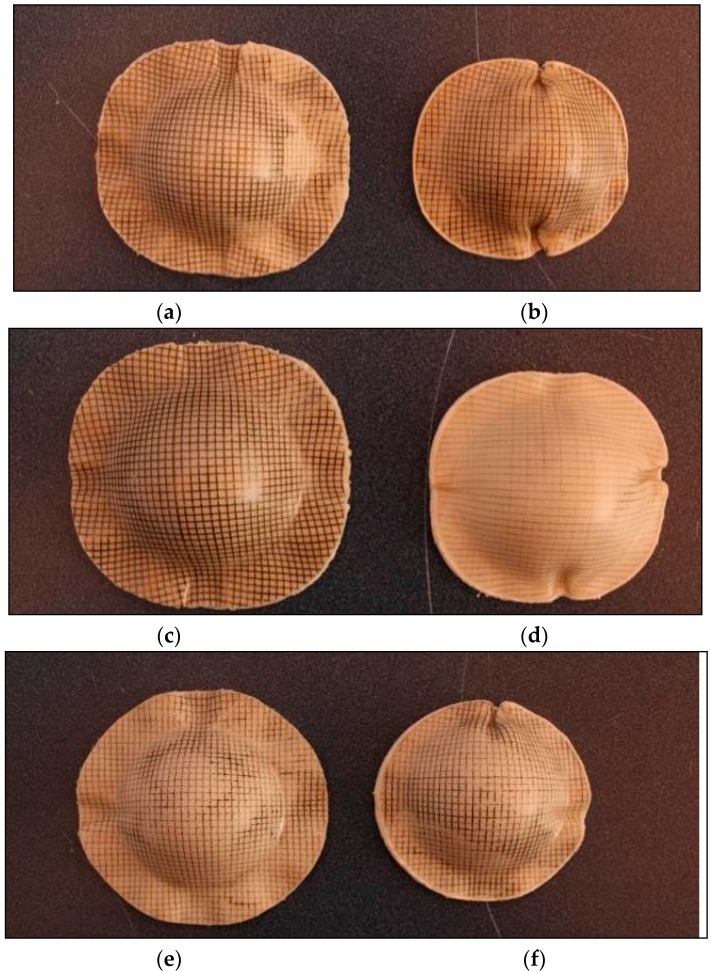
Domes formed from bamboo fabric-PLA composites with different layups and blank sizes. (**a**) Warp, 60 mm/min, 90 mm; (**b**) Warp, 60 mm/min, 80 mm; (**c**) w/f/f/w, 60 mm/min, 90 mm; (**d**) w/f/f/w, 60 mm/min, 80 mm; (**e**) w/45/−45/w, 60 mm/min, 90 mm; (**f**) w/45/−45/w, 60 mm/min, 80 mm.

Figure 11 presents the bamboo fabric-PLA composites with different layups after forming. The results show that the flange outline contours of the specimens change during forming according to the fabric layups. In the first phase of forming, the male die or punch touches the centre of the laminate, pushing it down into the female cavity. This means, that the initially flat laminate is shaped by draping it onto the surface of the male die. From Figure 11, it is obvious that the diameter of the circular laminate in the warp and weft directions is reduced after forming due to the inextensibility of the reinforcing fibres. As the male die moves towards its matching counterpart, the outer regions of the laminate have to travel towards the centre in order to fit the cavity of the mould [6].

In order to achieve satisfactory die conformity, the laminate has to alter its shape through inter-ply slip and more importantly, inter-ply rotation and intra-ply shear (trellis action). Trellis action occurs when the orthotropic characteristics of plain woven composites allow large shear deformation of square units in the fabric. It is the primary mechanism of textile forming. These square units can be stretched in the 45° direction associated with contraction in the normal direction, which reduces the wrinkling effect during the deformation process [14].

The trellis mechanism continues until the shear angle becomes high. The in-plane shear stiffness of a fabric increases intensely as the shear angle becomes large, particularly when it reaches and surpasses the “locking angle” at which lateral contact occurs between adjacent yarns. This increase in shear stiffness leads to wrinkling onset. Typically, the maximum fabric wrinkling occurs in the 45° direction [14]. The significant reduction of fibre crossing angle in the 45° direction is required to accommodate the large elongation and to smooth the wrinkled fabric through stretching in that direction [11,26].

Figure 11 displays the fact that the maximum fabric wrinkling occurs near to the fibre directions (in both warp and weft directions) for all laminates regardless of their layups. This finding contradicts the results reported in the literature [14], in which the maximum wrinkling was found to occur in the 45° direction. This observation may be due to the fact that though in-plane shear stiffness plays a large role in the onset of wrinkles in double-curved shape forming, there is no direct relation between shear angle and wrinkling. Wrinkling is a global phenomenon that depends on the set of all strains and stiffnesses.

Bending stiffness also plays an important role in wrinkle shape and patterns [27]. In the forming of thin textile composite reinforcements, the thickness is small compared to the warp and weft lengths. These thin structures tend to wrinkle and this phenomenon is exacerbated by the fibrous nature of the reinforcement as its bending stiffness is much lower than its in-plane stiffness. The bending stiffness of a woven fabric is also less than that of a laminate made from a continuous material because of sliding between its constituent fibres. Thus, the ability to create wrinkles is very high in textile materials [27].

#### 3.3.1. Warp and w/f/f/w Laminates

The wrinkling pattern of the bamboo fabric composite laminates in this study can be correlated to the effect of the drapability of the fabric on wrinkling formations. This is because matched-die forming shapes a laminate by draping it onto a male die [8]. Research by Hu [28] stated that the phenomenon of wrinkle formation is one of buckling and post-buckling deformations in terms of structural mechanics. Fabric sheets are very flexible in bending and can buckle easily under compressive stress, leading to wrinkle formation. In that study, two square pieces of fabric, one of wool and one of cotton, were draped over a sphere of 5 cm in radius in order to compare their drapability. The results from their experiment showed that both fabrics feature four main folds with smaller curved wrinkles between them as indicated by Hu [28]. These observations are almost identical to the results obtained in the current study (Figure 10) for the warp and w/f/f/w laminates, which showed four main wrinkles occurring parallel to the yarn directions.

**Figure 12:**
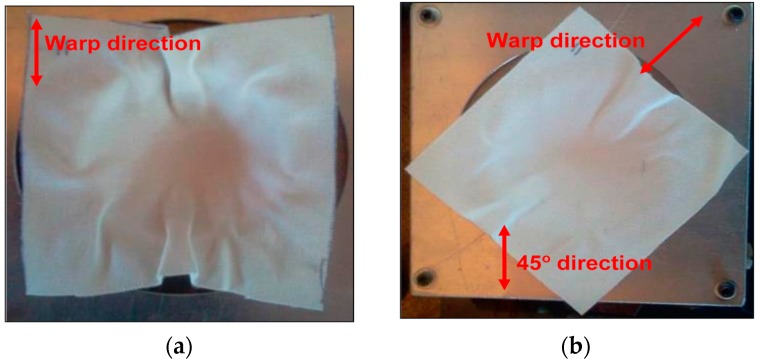
Formation of wrinkles in a dry bamboo fabric for (**a**) warpand; (**b**) 45° directions.

The other cause of wrinkling is the bending stiffness, which mainly determines the size and shape of wrinkles in the woven fabric. A higher bending stiffness leads to an increase in the size of wrinkles [27]. The bamboo fabric used in this study is an unbalanced fabric. It can be expected that warp direction will have a higher bending stiffness as it has more yarns compared to the weft direction. Figure 12 shows the formation of wrinkles in the dry bamboo fabric; the wrinkles in the warp direction are remarkable in comparison to the weft direction when a dry bamboo fabric layer was draped over a hemisphere of 25 mm in radius. In the warp direction, which possesses higher rigidity compared to weft direction, substantial fabric sliding can be seen. In contrast, no edge movement is depicted in the weft direction as reported in the literature [27].

The results in Figure 11 indicates that the wrinkles in the warp direction are remarkable in comparison to the weft direction particularly in specimens of 80 mm diameter, which are in fairly good agreement with Figure 12. The other significant observation of the warp laminate after forming can be seen in the flange area of the part; the material is only drawn into the die in the direction of the reinforcements, changing the circular blank to a dome that appears closer to a square shape in top view. Figure 11 shows that warp laminates deform equally in the 45° direction.

Laminates of w/f/f/w exhibited similar behaviour to the warp laminates. Although the warp direction consists of more yarns (2 yarns) compared to the weft direction (1 yarn), the effect of this difference is very small, particularly in specimens with larger diameters as can be seen in Figure 11. This is because the warp yarn has lower yarn thickness compared to weft yarn. Thus, lead to the small difference between all warp and w/f/f/w laminates. As with the warp laminate behaviour, the other notable observation of the w/f/f/w laminate after forming can be seen in the flange area of the formed part; the material is only drawn into the die in the direction of the reinforcements, changing the circular blank to a dome that appears closer to a square shape in top view. As with the warp laminates, Figure 11 also displays that w/f/f/w laminates deformed equally in the 45° direction.

#### 3.3.2. w/45/−45/w Laminates

In the case of w/45/−45/w laminates, the surface plies were arranged in the warp direction whereas subsurface plies were arranged in the ±45° direction. It was assumed that individual plies deform independently of each other. Previous experiments involving circular blanks with ±45° plies resulted in eight equal-sized buckles along a fibre direction, transverse to a fibre direction and at ±45° to a fibre direction [9]. However, in the current study, the results for PLA composite laminates indicated that the wrinkles were significant in the warp and weft directions, with small wrinkles in the ±45° directions. This wrinkle pattern may be because circular fabric sheets over circular mould are sensitive to any initial imperfections or deviations. These deviations are the result of more than two fibre orientations being present in the laminates. Different wrinkle patterns may appear in repeated experiments using the same fabric sheet on the same mould as reported by Hu [28].

A remarkable phenomenon observed when forming laminates with similar blank shape but different layups is shown in Figure 11. Depending on the arrangement of the reinforcements, the outline of the formed dome-shaped components differs noticeably. It is interesting to note that the final shape obtained with w/45/–45/w is different from that of the warp and w/f/f/w laminates. The shape of w/45/–45/w laminate is more circular compared to warp and w/f/f/w. This phenomenon can be correlated with the work done by Cherouat and Billoet [29]. They described that final shape obtained with 0°/90° fibre orientation is different from that obtained with a ±45° direction. Sadighi et al. [30] also reported that laminates with ±45° stacking sequence yield a final product outline with a very circular shape because resistance against forming is distributed almost equally in radial directions.

It is noteworthy that at high forming rates, severe wrinkling and buckling was observed in the w/45/−45/w laminate. This is because in this laminate, the 45° directions are not parallel to each other. The 45° deformation of one ply is blocked by the stiffer fibres in the other ply and vice versa. The loads are transferred between the individual plies by the interface adhesions. As a consequence, more wrinkling occurs [5]. This demonstrates that a change in layup orientation significantly affects the formability.

### 3.4. Effect of Blank Size (Forming Ratio)

In order to consider the effects of process parameters on double-curvature deformation, it is also necessary to define a measure of formability. In the case of single curvature deformation, formability has been evaluated in terms of shape fixability. For double-curvature deformation, formability can be assessed by considering the maximum extent of deformation possible for a given blank size. Hence, the limiting forming ratio for matched-die forming of a hemisphere from a circular blank is defined as the largest ratio of blank-to-dome area (A_blank_/A_dome_) that may be successfully formed:

(2) $$\text{Forming ratio}=\frac{\text{blank area}}{\text{formed dome area}}=\frac{{\pi r}_{0}^{2}}{2{\pi r}_{dome}h}=\frac{r_{0}^{2}}{2r_{dome}h}$$

Where *h* = maximum depth of the dome, *r*_0_ = blank radius, *r_dome_* = dome radius, which is equal to the die cavity radius, *r_die_*.

Table 3 indicates that the forming ratio calculated for blanks with diameters of 80 mm and 90 mm are 1.28 and 1.62, respectively. It can be seen from Figure 11 that the samples with a forming ratio of 1.62 showed no buckling within the dome area, while the samples with a forming ratio of 1.28 exhibited some buckling within the dome area. Decreasing the blank size appears to have a significant influence on buckling. Therefore, the limiting forming ratio for PLA composites is found to be 1.62 as severe buckling occurs within the useful part of the samples for blanks with lower size (1.28 forming ratio).

This phenomenon contradicts the results reported by Friedrich [8] and Hou [11]; they concluded that a larger blank size produces buckling in the flange area and suggested that buckling can be caused by the excess material, which remains outside the formed area. O’Bradaigh and Bryan [31] reported that an increase in preform size will lead to a greater area of resistance to the movement in the fibres and thus greater compressive stress. The current observation might be due to the fact that with a small blank size (80 mm), the flange area was small and after drawing there was sometimes not sufficient material to cover the entire die surface area. Bhattacharyya et al. [17] supported this finding, stating that the blanks that are smaller than the optimised size can also result in incomplete and unacceptable products because the matched-die forming tends to produce a more uniform thickness distribution. Buckling not only occurred in the flange area but also in the useful part area. Thus, in the case of the reinforcement used in this study, 90 mm was considered a suitable blank size to form good dome shapes. It is likely that increasing the blank size of PLA composites higher than the forming ratio of 1.62 will result in buckling in the flange area due to the reasons as suggested in the literature [8,11,31].

### 3.5. Strains Occurring During Matched-Die Thermoforming

**Figure 13:**
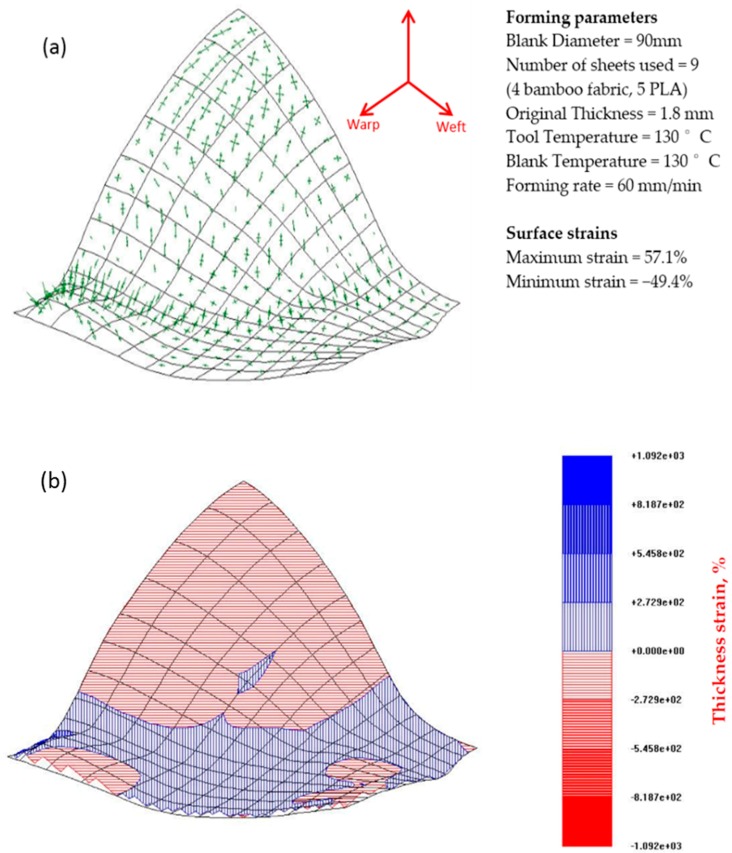
(**a**) Arrow diagram showing the surface strain map and (**b**) thickness strain contour plot for the bamboo fabric-PLA composite laminate of w/f/f/w layup formed using hot tooling conditions. Errors (mm): from 342 points, max = 2.54 Avg = 0.33.

**Figure 14:**
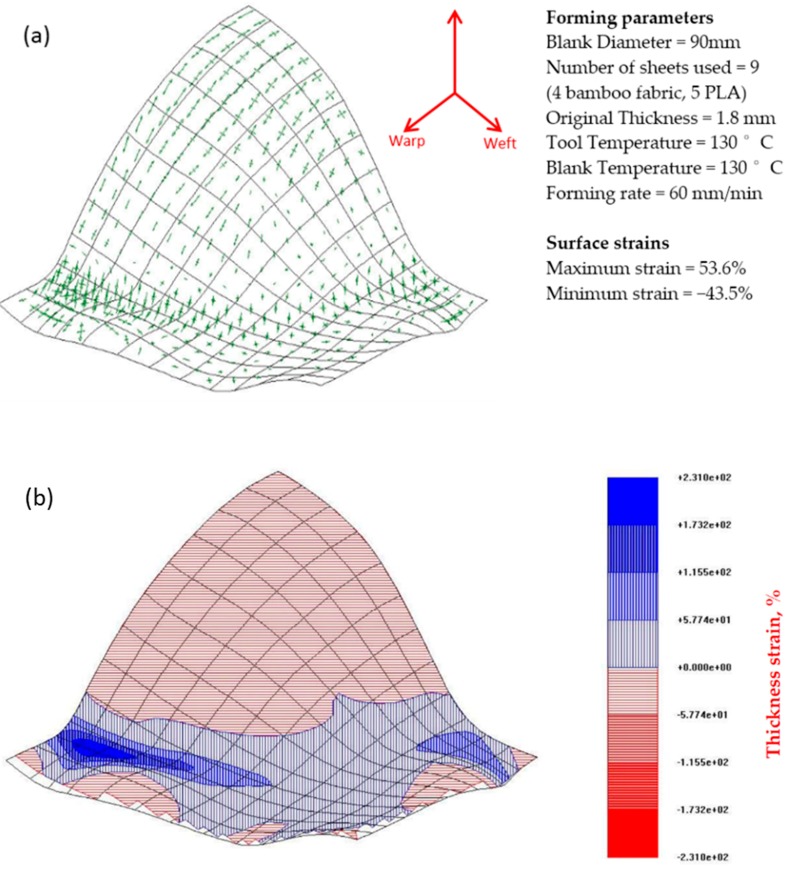
(**a**) Arrow diagram showing the surface strain map and (**b**) thickness strain contour plot for the bamboo fabric-PLA composite laminate of w/45/−45/w layup formed using hot tooling conditions. Errors (mm): from 387 points, max = 2.16 Avg = 0.321.

Figure 13 and Figure 14 depict the strain distribution and thickness changes in the deformed surfaces of the w/f/f/w and w/45/−45/w laminates of bamboo fabric-PLA composites. The results show that both laminates thicken in the flange area and the base of the dome along with a clear strain gradient moving towards the apex of the dome. The arrow diagrams showing the surface strain of the composites indicate that large strains have occurred in the laminates. The figures also show unbalanced biaxial strain states at the apex of the domes. The thickness strains confirm this, showing larger maximum thickness strains in the laminates.

Large strains were observed in the warp direction, while smaller strains can be seen in the weft direction for both w/f/f/w and w/45/−45/w laminates. This observation matched well with the images of composites that showed larger wrinkles in the warp direction and smaller wrinkles in the weft direction (Figure 11). Remarkable thickening was also observed in the warp direction as indicated in the thickness contour plot of the composites. As the GSA software does not cope well with specimens that have defects such as buckling and wrinkling, very large maximum thickness changes for the laminate in the warp direction was recorded. This may be due to excessive stretching occurring in localised regions and can be reduced by increasing the laminate thickness as suggested by Martin et al. [32]. As mentioned earlier, the thickness of the composites is approximately 1.8 mm. The lower thickness most likely affects the formability of the composites due to the occurrence of transverse flow during the forming. Transverse flow is observed during forming at elevated forming temperatures due to the local pressure gradients that arise from small variations in the laminate thickness and mould clearance. Transverse flow can also result from shear stresses developing between the thermoplastic material and the forming tool surface. The bending stiffness in the warp direction is higher than that of weft direction, causing the fabric to slide more and increase the strain magnitudes in this direction as shown in Figure 13 and Figure 14.

## 4. Conclusions

The purpose of the experiment discussed here is to investigate the effect of different parameters on the occurrence of deformations during sheet forming of double curvature shapes with bamboo fabric-PLA composites. The effect of unbalanced woven fabrics on the thermoformability of the composite laminate was studied. Further analysis was also carried out to investigate the behaviour of laminates with different blank sizes and different fabric stacking sequences. To obtain a physical measure of the specimen’s behaviour, the Grid Strain Analysis (GSA) technique was used to investigate the surface and thickness strains in the material.

Bamboo fabric-PLA composites exhibited less wrinkles and buckles under a low forming rate. The domes formed using hot tooling conditions were significantly better in quality than samples formed using cold tooling conditions. Wrinkles were more notable in the warp direction compared to weft direction. The results indicate that a laminate formed using a 90 mm blank with 130 °C die temperature, 130 °C blank temperature and 60 mm/min forming rate results in a well formed part with good conformity with the die. However, all specimens had small wrinkles at the flange region. For some forming processes, there are zones where wrinkles cannot be avoided. 

Depending on the arrangement of the fabrics, the outline or shape of the dome-shaped components differs noticeably. For warp and w/f/f/w laminates, the initially circular blank deforms into square shape, while the initially circular shape of w/45/−45/w laminate blank remains the same after the forming process. Thus, it becomes obvious that there is a close link between the laminate architecture and the resulting outline of the thermoformed component. The size of the blank and orientation of the fabric layers are the important factors need to be considered to achieve a well-formed component without buckles or wrinkles in their useful part.

One of the significant findings of this work is the difference in the formability behaviour of the unbalanced fabric used in this study. Unbalanced fabric has different bending stiffness in the warp and weft directions, resulting in variations in wrinkle shape and size. The in-plane shear stiffness plays a pivotal role in the onset of wrinkling in double-curved shape forming, although there is no direct relation between shear angle and wrinkling. Bending stiffness also plays an important role in the wrinkle shape and patterns.

The GSA technique provides a snapshot of the deformation process, which illustrates the characteristic surface and thickness strains generated during the forming of the composite sheets. GSA also identifies the regions of severe deformation and provides useful information regarding the optimisation of processing parameters, hence helping to improve the quality of the product.

## Figures and Tables

**Table 1:** Mechanical and physical properties of PLA (according to the datasheet).

Material	PLA
Trade name	Ingeo biopolymer 2003D
Density (g/cm^3^)	1.24
Tensile stress (MPa)	53
Flexural modulus (MPa)	–
Notched izod impact strength (23 °C) (J/m)	16
Falling dart impact (23 °C) (J)	–
Heat distortion temperature (°C)	55
Melt mass flow rate (MFR) (230 °C/2.16 kg) (g/10 min)	6

**Table 2:** Dome forming test parameters for bamboo fabric-PLA composites using cold and hot tooling conditions.

Tooling Conditions	Test Parameters for Bamboo Fabric-PLA Composites				
	Blank Temperature (°C)	Die Temperature (°C)	Forming Rate (mm/min)	Blank Size (mm)	Orientation of the Plies
Cold tooling conditions	155	23	60	90	warp
					w/f/f/w
					w/45/−45/w
			100		warp
					w/f/f/w
					w/45/−45/w
Hot tooling conditions	130	130	60	90	warp
					w/f/f/w
					w/45/−45/w
				80	warp
					w/f/f/w
					w/45/−45/w
	130	130	40	90	w/f/f/w
			60		
	140		60		
	100	130	60	90	w/45/−45/w
	130				

**Table 3:** Forming ratio (FR) calculated for samples in Figure 11 using Equation (1).

Specimen	Max Dome Depth (*h*) (mm)	Cavity Rim Radius (*r_rim_*)	Blank Radius (*r*_0_) (mm)	Dome Radius (*r_dome_*) (mm)	Blank Area (A_0_) (mm^2^)	Dome Area (A_1_) (mm^2^)	Forming Ratio (FR)
Diameter 80 mm	25	25	40	25	5027	3928	1.28
Diameter 90 mm	25	25	45	25	6362	3928	1.62
